# Generation and analysis of blueberry transcriptome sequences from leaves, developing fruit, and flower buds from cold acclimation through deacclimation

**DOI:** 10.1186/1471-2229-12-46

**Published:** 2012-04-02

**Authors:** Lisa J Rowland, Nadim Alkharouf, Omar Darwish, Elizabeth L Ogden, James J Polashock, Nahla V Bassil, Dorrie Main

**Affiliations:** 1 Genetic Improvement of Fruits and Vegetables Laboratory, USDA-ARS, BARC-West, 10300 Baltimore Ave., Beltsville, MD 20705, USA; 2 Department of Computer and Information Sciences, Towson University, 8000 York Road, Towson, MD 21252, USA; 3 Genetic Improvement of Fruits and Vegetables Laboratory, USDA-ARS, Blueberry and Cranberry Research Center, 125A Lake Oswego Road, Chatsworth, NJ 08019, USA; 4 National Clonal Germplasm Repository, USDA-ARS, 33447 Peoria Road, Corvallis, OR 97333-2521, USA; 5 Department of Horticulture and Landscape Architecture, Washington State University, Pullman, WA, USA

## Abstract

**Background:**

There has been increased consumption of blueberries in recent years fueled in part because of their many recognized health benefits. Blueberry fruit is very high in anthocyanins, which have been linked to improved night vision, prevention of macular degeneration, anti-cancer activity, and reduced risk of heart disease. Very few genomic resources have been available for blueberry, however. Further development of genomic resources like expressed sequence tags (ESTs), molecular markers, and genetic linkage maps could lead to more rapid genetic improvement. Marker-assisted selection could be used to combine traits for climatic adaptation with fruit and nutritional quality traits.

**Results:**

Efforts to sequence the transcriptome of the commercial highbush blueberry (*Vaccinium corymbosum*) cultivar Bluecrop and use the sequences to identify genes associated with cold acclimation and fruit development and develop SSR markers for mapping studies are presented here. Transcriptome sequences were generated from blueberry fruit at different stages of development, flower buds at different stages of cold acclimation, and leaves by next-generation Roche 454 sequencing. Over 600,000 reads were assembled into approximately 15,000 contigs and 124,000 singletons. The assembled sequences were annotated and functionally mapped to Gene Ontology (GO) terms. Frequency of the most abundant sequences in each of the libraries was compared across all libraries to identify genes that are potentially differentially expressed during cold acclimation and fruit development. Real-time PCR was performed to confirm their differential expression patterns. Overall, 14 out of 17 of the genes examined had differential expression patterns similar to what was predicted from their reads alone. The assembled sequences were also mined for SSRs. From these sequences, 15,886 blueberry EST-SSR loci were identified. Primers were designed from 7,705 of the SSR-containing sequences with adequate flanking sequence. One hundred primer pairs were tested for amplification and polymorphism among parents of two blueberry populations currently being used for genetic linkage map construction. The tetraploid mapping population was based on a cross between the highbush cultivars Draper and Jewel (*V. darrowii *is also in the background of 'Jewel'). The diploid mapping population was based on a cross between an F_1 _hybrid of *V. darrowii *and diploid *V. corymbosum *and another diploid *V. corymbosum*. The overall amplification rate of the SSR primers was 68% and the polymorphism rate was 43%.

**Conclusions:**

These results indicate that this large collection of 454 ESTs will be a valuable resource for identifying genes that are potentially differentially expressed and play important roles in flower bud development, cold acclimation, chilling unit accumulation, and fruit development in blueberry and related species. In addition, the ESTs have already proved useful for the development of SSR and EST-PCR markers, and are currently being used for construction of genetic linkage maps in blueberry.

## Background

Blueberry (*Vaccinium *section *Cyanococcus*) is an economically important small fruit crop adapted to acidic, sandy soils that otherwise might be considered worthless from an agronomic standpoint [1]. Blueberry is a member of the *Ericaceae *family, which includes many acid-loving species, such as the commercially important berry crops, blueberry, cranberry, and lingonberry, and the floral and nursery crops, rhododendron, azalea, and mountain laurel. The commercial blueberries are derived principally from four species, the tetraploid highbush blueberry (*Vaccinium corymbosum*), the diploid and tetraploid lowbush blueberry (*V*. *myrtilloides *and *V. angustifolium*, respectively), and the hexaploid rabbiteye blueberry (*V. virgatum*), and hybrids thereof. North America is the major producer of blueberries, although production of blueberries is on the rise worldwide. About 2/3 of blueberry production in the U.S. is from improved cultivars of the tetraploid highbush blueberry (*V. corymbosum*) and about 1/3 is from wild, managed fields of the tetraploid lowbush blueberry (*V. angustifolium*) [2].

There has been increased demand for and consumption of blueberries in recent years because of their many recognized health benefits. Blueberry fruit is very high in anthocyanins, which have been linked to improved night vision, prevention of macular degeneration, anti-cancer activity, and reduced risk of heart disease [3,4]. The compound resveratrol, found in blueberries, has been linked to reduced risk of heart disease and cancer, and another compound, pterostilbene, has been shown to lower cholesterol [5].

Much progress has been made in traditional breeding of highbush and rabbiteye cultivars since their domestication in the early twentieth century. Breeding efforts have focused on development of cultivars with broader climatic adaptation (increased freezing tolerance for northern regions and reduced chilling requirements for southern regions), broader soil adaptation (ability to grow in higher pH soils), disease and pest resistance, mechanical handling tolerance, and high fruit quality [1]. Lowbush blueberry, on the other hand, is a managed wild crop, and little effort has been made to breed improved varieties. Improved cultural practices, however, have resulted in dramatic increases in yields of lowbush blueberry over the last two decades [6,7].

Until now, few genomic resources have been available for blueberry, or for the *Ericaceae *family in general. The availability of genomic tools for molecular breeding could possibly lead to more rapid genetic improvement of blueberry, particularly when combining traits for climatic adaptation with other important traits like fruit and nutritional quality. Blueberry is especially suitable for improvement via marker-assisted breeding because of its long generation time, high heterozygosity, self-infertility especially of diploids, inbreeding depression, and polyploidy of commercial types. In the last 6-7 years, the first few thousand expressed sequence tags (ESTs) were generated and made publicly available for this family, about 5,000 from blueberry [8,9] and about 1,200 from rhododendron [10]. A limited number of robust molecular markers like simple sequence repeats (SSRs) [11] and expressed sequence tag-polymerase chain reaction markers (EST-PCRs) [12-14] were developed from some of the publicly available blueberry ESTs and are being used in genetic diversity and mapping studies. Current mapping studies are focused on identifying quantitative trait loci (QTL) associated with chilling requirement, freezing tolerance, and fruit quality traits in blueberry. The first microarray experiments have been carried out in blueberry and have successfully identified many transcripts whose abundances increase with cold acclimation [9,15]. More gene expression studies need to be undertaken, however, that are based on a larger collection of gene sequences in order to sort out genes that are expressed in response to various stimuli and during development.

Because freezing tolerance (especially of flower buds) and fruit quality are important traits that could be improved upon using marker-assisted-selection, we report here the generation and analysis of a large collection of publicly available ESTs from flower buds at different stages of cold acclimation, fruit at different stages of ripening, and leaves of highbush blueberry using a high-throughput pyrosequencing approach, based on Roche's 454 Genome Sequencer (GS) FLX Titanium platform. Next generation 454 EST sequencing has been shown to be a very efficient, cost-effective approach for transcriptome analysis of non-model species and for the discovery of rare and novel transcripts [16-21]. In our study, over 600,000 reads were assembled into approximately 15,000 contigs and 124,000 singletons. The contigs and singletons have been annotated and functionally mapped to Gene Ontology (GO) terms. A database was developed to house these sequences and their annotations, and a web-based interface was developed to allow others to search/browse the data http://bioinformatics.towson.edu/BBGD454. In addition, the frequency of the most abundant sequences in each of the libraries was compared across all libraries to identify genes that are potentially differentially expressed during cold acclimation and fruit development. Differential expression of most of these genes was confirmed through real-time PCR. The assembled ESTs were also screened for simple sequence repeat (SSR) motifs in order to design SSR primers for ongoing mapping studies. These sequences constitute an important genomic tool for the scientific community, particularly for those interested in gene discovery, expression, and mapping/relationship studies in the *Ericaceae *family.

## Results and discussion

### 454 EST sequencing and assembly

Nine cDNA libraries were constructed from mRNA from various organs collected from plants of the highbush blueberry (*V. corymbosum*) variety Bluecrop. Organs included young, fully expanded leaves, flower buds collected at various stages of cold acclimation (0, 397, 789, and 1333 chill units), and fruit collected at various stages of ripening (green, white, pink, and blue). From many years of cold hardiness research, we have shown that 'Bluecrop' flower buds (from plants grown in the Mid Atlantic region) have a cold hardiness level or LT_50 _of about -13°C in late September or early October (0 chill units). Cold hardiness reaches a maximum level (or minimum LT_50_) of about -25 to -27°C by about mid to late December (~600 chill units), and buds begin to deacclimate in February and reach a cold hardiness level of about -13 to -14°C by late March (~1300 chill units) [22,23]. Thus, the four flower bud libraries corresponded to time points when (1) flower buds were being formed and large enough to first collect--here plants were still essentially non-acclimated or in the very early stages of acclimation (0 chill units), (2) plants were approaching maximum cold hardiness (397 chill units), (3) plants had reached and were maintaining maximum cold hardiness (789 chill units), and (4) plants had nearly completely deacclimated and buds were beginning to open (1333 chill units).

**Figure 1:**
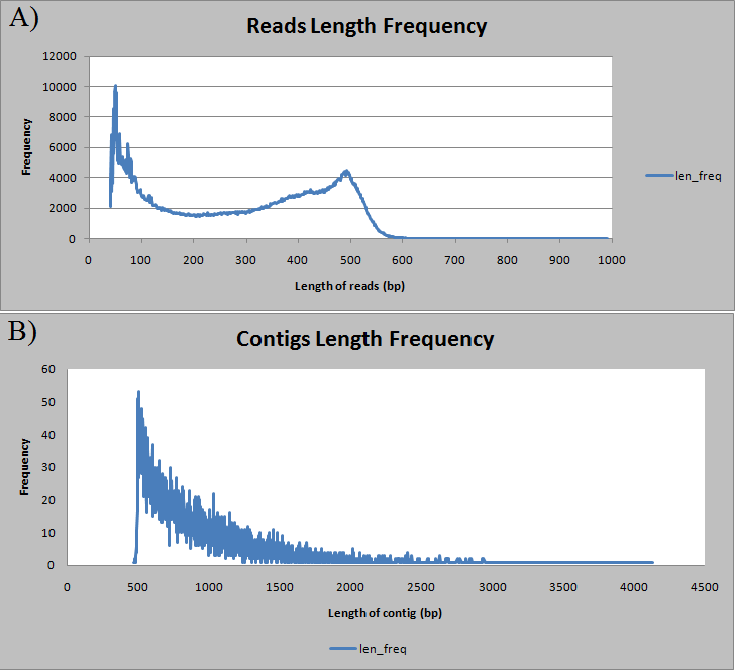
**Distribution of read and contig lengths from the *V. corymbosum *454 transcriptome sequencing project**. A. Length distribution of 454 sequencing reads before removal of sequences below 50 nt. B. Length distribution of contigs after assembly of all the 454 sequences (including removal of low quality sequences and sequences below 50 nt in length).

The nine libraries (leaves, buds at four different stages of cold acclimation, and fruit at four different stages of ripening) were multiplexed and sequenced on two plate runs of the 454-GS FLX Titanium platform. A summary of the sequencing and assembly results is shown in Table 1. Overall, 1,348,819 reads were generated, with an average read length of 287 nucleotides (nt). This yielded a total of ~390 megabases of cDNA sequence. The length distribution for the reads is shown in Figure 1A. Before removal of any low quality sequences or sequences that were too short, 64% of the reads had lengths in the 200-600 nt range.

**Table 1:** Summary of 454 blueberry EST data

Sample	Total number of reads assembled	Total number of reads in contigs	Number of contigs/singletons	Average contig/singleton length (nt)^a^
Flower bud 0'^b^	69,943	43,073	2,675/26,870	804/323

Flower bud 397'	74,169	39,999	2,751/34,170	760/306

Flower bud 789'	69,874	37,681	2,645/32,193	785/319

Flower bud 1333'	72,733	41,836	2,421/30,897	796/302

**All flower bud samples**	**291,342**	**228,938**	**10,350/62,404**	**898/280**

Green fruit	73,168	46,708	2,241/26,460	720/284

White fruit	69,260	42,682	2,029/26,578	700/298

Pink fruit	68,767	43,975	1,964/24,792	750/297

Blue fruit	59,622	37,615	1,941/22,007	819/311

**All berry samples**	**259,527**	**199,643**	**6,726/59,884**	**818/267**

Leaves and stems	62,465	36,763	1,781/25,702	771/298

**All samples**	**614,028**	**490,517**	**14,764/123,511**	**933/253**

^a^nt = nucleotides

^b^hours refer to hours of low temperature exposure

**Figure 2:**
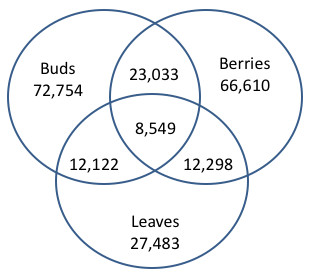
**Vinn diagram showing the total number of unigenes (contigs plus singletons) from each of three assemblies (leaves, buds, and berries) and the numbers of unigenes shared between each pair of assemblies and all three assemblies**.

Assembly of all the sequences, after removal of low quality sequences and sequences below 50 nt in length, resulted in a total of 138,275 unique putative transcript sequences--14,764 contigs, with an average length of 933 nt, and 123,511 singletons, with an average length of 253 nt. Contig length ranged from ~500-3000 nt, with the majority, by far, in the 500-2000 nt range (Figure 1B). Assembly of each of the bud libraries separately resulted in ~2,400-2,800 contigs and ~26,000-35,000 singletons, indicating that each of the libraries was of comparable quality. The assembly of all the bud sequences together resulted in 10,350 contigs and 62,404 singletons. Assembly of each of the berry libraries separately resulted in ~1,900-2,300 contigs and ~22,000-27,000 singletons, again indicating that each of the berry libraries was of comparable quality. Assembly of all the berry sequences together resulted in 6,726 contigs and 59,884 singletons. Assembly of the leaf sequences resulted in 1,781 contigs and 25,702 singletons; these numbers were in the same ballpark as each of the other libraries. Figure 2 shows the total number of unigenes (contigs plus singletons) from each of the three assemblies, leaf, bud, and berries, and the numbers of unigenes shared between each pair of assemblies and all three assemblies. Between the bud and leaf, the leaf and berry, and the berry and bud assemblies, 12,122, 12,298, and 23,033 unigenes were shared, out of a total of 100,237, 94,093, and 139,364 unique transcript sequences from each pairwise comparison. Among all three assemblies, only 8,549 unigenes were shared. These comparisons indicate that patterns of gene expression are quite different in the various organs examined, yielding a diverse array of transcript sequences.

### Annotation of sequences

Annotation of unique sequences (contigs and singletons) from all the various assemblies was attempted based on searches of specific databases for sequence similarity. Blast2Go [24], an annotation and visualization tool, was used to BLAST the contig and singleton sequences from each assembly against the non-redundant database (nr) of the National Center for Biotechnology Information (NCBI). Domain-finding tools, such as InterProScan [25], were also used to help annotate those sequences that had no good BLAST hits. Because of their greater length, the percentage of contigs from the 'all' assembly that had significant homology (E-values ≤ 10^-5^) to other sequences in GenBank was high (86.3%), much higher than the percentage of singletons with significant homology (18.5%). The percentage of these contig sequences that had significant homology to known plant proteins was 84.8%, whereas 1.5% had homology to unknown/hypothetical plant proteins and 13.7% had no significant homology to other sequences in GenBank. The number one species that the top BLASTX (search of the protein databases using a translated nucleotide query) contig searches hit was *Vitis vinifera *(grape), followed by *Ricinis communis *(the castor oil plant), *Populus tricocarpa *(black cottonwood) and *Glycine max *(soybean) in descending order. Of the various plant species for which we have at least a draft sequence available, *Vitis vinifera *is the most closely related to blueberry and is also a berry crop.

**Figure 3:**
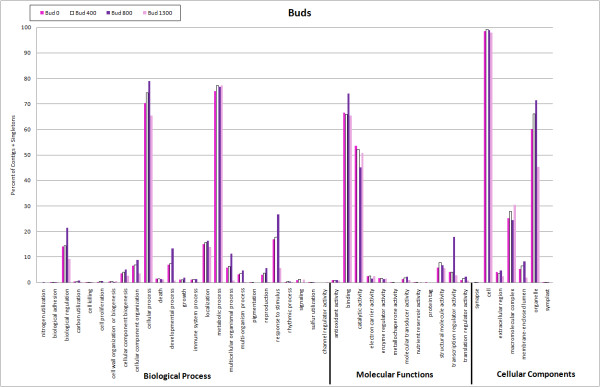
**Functional annotation based on GO categories of unigenes from the different flower bud assemblies (buds having received ~0, 400, 800, and 1300 chill units)**.

The sequences that showed significant similarity to annotated proteins in NCBI were assigned GO terms based on their associated biological processes, molecular functions, and cellular components. Figure 3 shows the categorization of sequences (contigs and singletons) from each of the bud assemblies. Under biological processes, the distribution of sequences from the first two bud libraries (0 and ~400 chill units), representing transcripts at early and middle stages of cold acclimation, were most similar. Most of the sequences were assigned to the metabolic process category, followed by cellular process, response to stimulus, localization, biological regulation, and developmental process/cellular component organization categories, in that order. The distribution of sequences from the third bud library (~800 chill units), which represents transcripts during the late stages of cold acclimation, when buds have had or nearly had their chilling requirements met, was somewhat different from the first two libraries. Most of the sequences were assigned to the cellular process category followed closely by metabolic process, then response to stimulus, biological regulation, localization, and developmental process. The distribution of sequences from the fourth bud library (~1300 chill units), which represents transcripts during deacclimation, was most different from the other three libraries. Most of the sequences were assigned to the metabolic process category, followed by cellular process, localization, biological regulation, response to stimulus, cellular component organization, and cellular component biogenesis. The high percentage of sequences in the metabolic process category and the lower percentage of sequences in the response to stimulus, biological regulation, and developmental process categories are perhaps consistent with the buds' deacclimating and resuming growth at this stage. Under molecular function, the top four categories for the three bud libraries (0, 400, and 1300 chill units) were binding, catalytic activity, structural molecule activity, and transcription regulator activity. For the 800 chill unit bud library, the top four categories were binding, catalytic activity, transcriptional regulator activity, and structural molecule activity. Under cell component, the top three categories for all four bud libraries were cell, organelle, and macromolecular complex.

**Figure 4:**
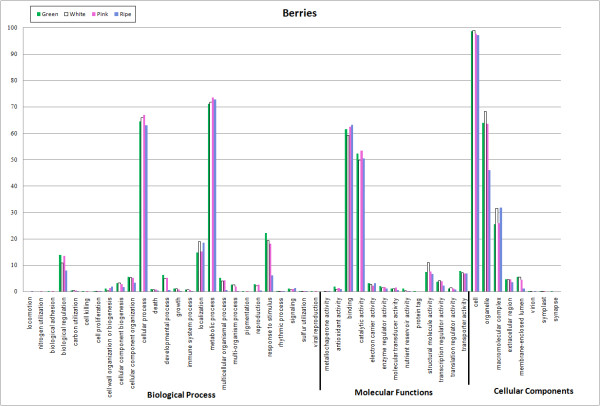
**Functional annotation based on GO categories of unigenes from the different berry assemblies (green, white, pink, and blue fruit ripening stages)**.

Figure 4 shows the categorization of sequences from each of the berry assemblies. As seen with the bud libraries, under biological process, the first three berry libraries (green, white, and pink fruit) were more similar to each other than to the fourth library (ripe or blue fruit). Similar to the first two bud libraries, most of the sequences from the first three berry libraries were assigned to the metabolic process category, followed by cellular process, response to stimulus, localization, biological regulation, and cellular component organization/developmental process, in that order. Most of the sequences from the ripe fruit library were assigned to the metabolic process category, followed by cellular process, localization, biological regulation, response to stimulus, and cellular component organization, as we saw with the deacclimating bud library. There were far fewer sequences in the response to stimulus, developmental process, and multicellular organismal/multi-organism process categories, than were found in the other three berry libraries. Under molecular function, the top categories for all four berry libraries were binding, catalytic activity, structural molecule activity/transporter activity, and transcription regulator activity/electron carrier activity. This was similar to the bud libraries, except that there were higher percentages of sequences in the transporter activity category in the fruit libraries than in the bud libraries. Under cell component, the top three categories for all four berry libraries, as with the four bud libraries, were cell, organelle, and macromolecular complex.

**Figure 5:**
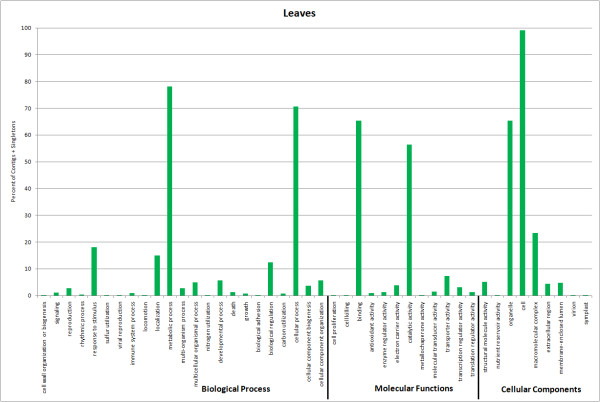
**Functional annotation based on GO categories of unigenes from the leaf assembly**.

Figure 5 shows the categorization of sequences from the leaf assembly. Most of the sequences were assigned to the metabolic process category, followed by cellular process, response to stimulus, localization, biological regulation, and cellular component organization/developmental process, in that order. This was similar to the first two bud libraries and first three berry libraries. Under molecular function, the top six categories for the leaf library were binding, catalytic activity, transporter activity, structural molecule activity, electron carrier activity, and transcription regulator activity. This was quite similar to the berry libraries. Under cell component, the top three categories were cell, organelle, and macromolecular complex, as with all the other libraries.

### Comparison of sequence abundance across libraries and real-time PCR results

In addition to comparing the libraries based on annotation of the sequences, the most highly abundant transcripts (contigs with the most number of reads) were identified in each of the libraries. Their sequences were then BLASTed against the other eight libraries to determine the number of homologous reads in each of the other libraries. In this way, highly abundant transcripts that were potentially differentially expressed during cold acclimation and during fruit development were identified. Additional file 1 lists the ~30 most highly abundant transcripts from all nine libraries and their total numbers of reads (and percentages of total reads) across each of the libraries. In the last columns, we give our predictions, from the percentages of reads, as to whether each of the transcript levels goes up, down, up then down, down then up, etc., or stays fairly constant across the four bud time points/stages of cold acclimation (0, 397, 789, and 1333 chill units) and the four stages of fruit development (green, white, pink, and blue).

From Additional file 1, it is clear that many of the transcripts appear to be differentially expressed during cold acclimation. Levels of most of the highly abundant transcripts from the Bud 0' library appeared to either decline as cold acclimation progressed and then rise again during deacclimation (down/up designation), or to decline at all the time points past the 0' point (down). Levels of many of the most highly abundant transcripts from the Bud 397' and Bud 789' libraries appeared to rise during acclimation (from 0' to 397' or from 0' to 789') and then decline during deacclimation (1333'). Thus, they were given an up/down designation. Levels of many of the most highly abundant transcripts from the Bud 1333' library appeared to decline during cold acclimation and then rise again during deacclimation (down/up), as with the Bud 0' library. Levels of about 1/3 of the top 30 most highly abundant transcripts in the Bud 1333' library appeared to be low at all the time points prior to deacclimation and then to rise sharply during deacclimation (up).

These results were very similar to what we found previously using a small microarray to identify differentially expressed transcripts during cold acclimation/deacclimation [9]. Although the microarray was based on only about 1,500 unique genes [9,15], many of the transcripts identified as differentially expressed in that study were also identified as potentially differentially expressed in this study. Some of the transcripts identified as potentially downregulated during cold acclimation in both studies encoded dehydration-induced RD22-like protein/BURP domain-containing protein, certain heat shock proteins, certain dehydrins, beta-glucosidase, UDP-glucose dehydrogenase, and ascorbate peroxidase (Additional file 1 and [9]). Transcripts identified as potentially upregulated during cold acclimation in both studies encoded DNA-binding domain proteins, certain LEAs (late embryogenesis abundant)/dehydrins, protease inhibitors, various ribosomal proteins, galactinol synthase, granule-bound starch synthase, and S-adenosylmethionine decarboxylase (Additional file 1 and [9]). Obtaining similar results from the microarray study and the deeper transcriptome sequencing study suggests that this new database will be very useful for further elucidating the cold acclimation response pathway in blueberry.

Furthermore, this study allowed identification of more potentially differentially expressed transcripts than were discovered in the microarray study, because of the deeper sequencing done here. Many of the transcript sequences obtained in this study are new, so they were not included on the previous microarray. For example, from the comparison of reads across libraries, transcripts encoding pyrophosphate-dependent phosphofructokinase, arginine decarboxylase, lipoxygenase, abscisic stress ripening protein, and a hypothetical protein all appeared to be high at the earliest stage of cold acclimation (0' buds), then downregulated as cold acclimation progressed, then upregulated as deacclimation commenced, except for lipoxygenase which appeared to remain at low levels during deacclimation. On the other hand, transcripts encoding a lipid transfer protein, amino acid selective channel protein, Pointed First Leaf, and a high mobility group protein appeared to be low in 0' buds, upregulated as cold acclimation progressed, and then downregulated during deacclimation, except for Pointed First Leaf which actually appeared to peak during deacclimation. These genes are just a few examples of genes not identified previously in our microarray study as being differentially expressed.

**Figure 6:**
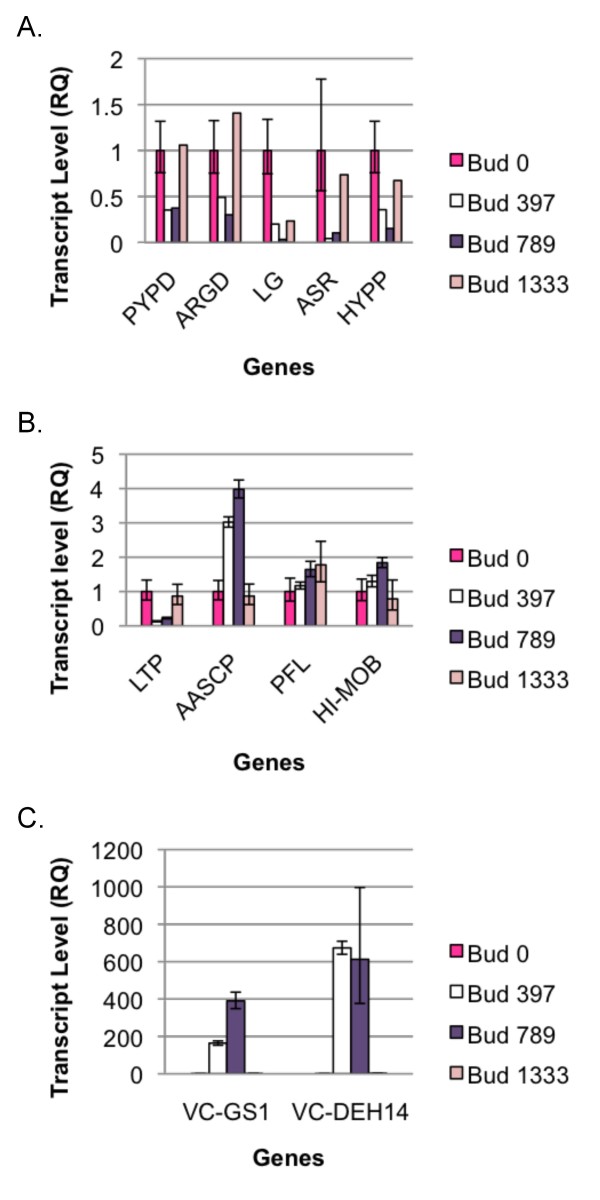
**Transcript levels from real-time PCR analysis of selected genes in flower buds that had received varying chill units (0, 397, 789, and 1333 chill units)**. A. Transcript levels of five genes (PYPD: pyrophosphate-dependent phosphofructokinase; ARGD: arginine decarboxylase; LG: lipoxygenase; ASR: abscisic stress ripening protein; HYPP: hypothetical protein) that appeared to be downregulated during cold acclimation based on their number of reads from each of the bud libraries. B. Transcript levels of four genes (LTP: lipid transfer protein; AASCP: amino acid selective channel protein; PFL: Pointed First Leaf; HI-MOB: high mobility group protein) that appeared to be upregulated during cold acclimation based on their number of reads from each of the bud libraries. C. Transcript levels of two genes (VC-GS1: galactinol synthase; VC-DEH14: 14 kDa dehydrin) that were already known to be upregulated in flower buds during cold acclimation [9,28].

Therefore, real-time PCR was performed to test whether these genes were indeed differentially expressed during cold acclimation (Figure 6). Figure 6A shows transcript levels across the four time points (0, 397, 789, and 1333') for five genes (pyrophosphate-dependent phosphofructokinase, arginine decarboxylase, lipoxygenase, abscisic stress ripening protein, and a hypothetical protein) that appeared to be downregulated during cold acclimation based on their number of reads. Indeed, all showed the expected trends in terms of their gene expression patterns, with all having high transcript levels at the earliest stage of cold acclimation (0' buds), being downregulated as cold acclimation progressed, then upregulated as deacclimation commenced, except for lipoxygenase which, as expected, remained at fairly low levels during deacclimation. Lipoxygenase, arginine decarboxylase, and abscisic stress ripening protein have all been reported to be associated with stress responses in other plants. Lipoxygenase, which catalyzes the oxygenation of free polyunsaturated fatty acids into oxylipins, is an important enzyme of the jasmonate signaling pathway, and has been shown to be associated with biotic and abiotic stress responses including response to low temperature [26]. A full-length lipoxygenase cDNA was recently cloned from apical buds of *Caragana jubata*, a plant that grows in extreme cold in the Himalayas [26]. A gene encoding arginine decarboxylase, a key enzyme responsible for polyamine synthesis under stress conditions, was recently isolated from *Poncirus trifoliata*. The gene was overexpressed in *Arabidopsis thaliana *and resulted in enhanced resistance to drought and cold stress. Accumulation of reactive oxygen species was decreased in the transgenic line [27]. A gene from lily encoding a member of the abscisic stress ripening (ASR) family of proteins was also recently overexpressed in *Arabidopsis*. Research on this protein and transgenic line suggests that the protein may act as an osmoprotectant as well as a transcription factor mediating cold/freezing stress signaling. Twelve genes appeared to be upregulated and 25 genes downregulated by expression of the lily ASR in *Arabidopsis *[28].

Figure 6B shows transcript levels across the four time points (0, 397, 789, and 1333') for four genes (lipid transfer protein, amino acid selective channel protein, Pointed First Leaf, and a high mobility group protein) that appeared to be upregulated during cold acclimation based on their number of reads, and Figure 6C shows transcript levels for two genes (galactinol synthase and 14 kDa dehydrin) that were already known to be upregulated in flower buds during cold acclimation [9,29]. Again, all showed the expected trends in gene expression, except for the gene encoding a lipid transfer protein. Instead of being upregulated during cold acclimation, its transcript level was higher in the early stages of cold acclimation, then downregulated as cold acclimation progressed, then upregulated during deacclimation. It is possible that the primers designed from its sequence may have had strong homology to another gene encoding a lipid transfer protein that is, in fact, downregulated during cold acclimation. Lipid transfer proteins have been found to be associated with cold acclimation ability in some *Solanum *species that are able to acclimate to cold [30]. Based on work done in *Arabidopsis*, Pointed First Leaf encodes a ribosomal protein S18. Activity of the S18A promoter appears to be restricted to meristems; plants activate an extra copy of this gene in tissues with cell division activity [31]. It is, therefore, notable that transcripts of Pointed First Leaf appear to peak during deacclimation of blueberry flower buds, when buds are resuming growth. Furthermore, expression of a gene encoding a chloroplastic amino acid selective channel protein has been correlated with cold acclimation in cereals [32].

From Additional file 1, it is also clear that many of the highly abundant transcripts appear to have a complex, differential expression pattern during fruit development. Levels of about half of the most highly abundant transcripts from the green and white fruit libraries appeared to either decline during fruit development or to decline initially and then rise again later during development. The other half appeared to rise, or rise and then decline, or rise, decline, and rise again. Levels of some of the transcripts appearing to decline throughout the ripening period encoded metallothionein-like, lipid transfer, dehydrin, ribulose-bisphosphate carboxylase oxygenase small subunit, and light harvesting complex II proteins. Levels of transcripts encoding a burp domain-containing protein and thioredoxin h appeared to decline until reaching the blue/ripe fruit stage, where they then rose sharply. Levels of transcripts encoding cysteine proteinase precursor appeared to rise during development and then decline at the blue/ripe fruit stage.

Levels of many of the most highly abundant transcripts from the pink fruit library appeared to rise, peaking at the pink fruit stage, and then to decline afterwards at the blue/ripe fruit stage. These included transcripts for pectate lyase, cytochrome b5, cysteine protease-like protein, cyclophilin, glutathione peroxidase, and chalcone synthase. Levels of many of the most highly abundant transcripts from the blue/ripe fruit library appeared to rise during fruit development, and peak at the blue/ripe fruit stage. These included transcripts for aspartic proteinase, flavonoid 3-hydroxylase, and 1-aminocyclopropane-1-carboxylate oxidase.

**Figure 7:**
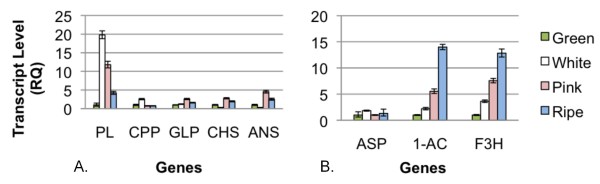
**Transcript levels from real-time PCR analysis of selected genes across varying stages of fruit ripening (green, white, pink, and blue/ripe fruit stages)**. A. Transcript levels of five genes (PL: pectate lyase; CPP: cysteine protease-like protein; GLP: glutathione peroxidase; CHS: chalcone synthase; ANS: anthocyanidin synthase) whose expression levels appeared to peak at the pink fruit stage based on their number of reads from each of the berry libraries. B. Transcript levels of three genes (ASP: aspartic proteinase; 1-AC: 1-aminocyclopropane-1-carboxylate oxidase; F3H: flavonoid 3-hydroxylase) whose expression levels appeared to peak at the blue fruit stage based on their number of reads from each of the berry libraries.

Real-time PCR was performed to verify that some of these genes were indeed differentially expressed during fruit development (Figure 7). Figure 7A shows transcript levels across the four development stages (green, white, pink, and blue) for five genes (pectate lyase, cysteine protease-like protein, glutathione peroxidase, chalcone synthase, and anthocyanidin synthase) whose expression levels appeared to peak at the pink fruit stage based on their number of reads. From real-time PCR, three of the genes, glutathione peroxidase, chalcone synthase, and anthocyanidin synthase, showed the expected trends in terms of their gene expression patterns, having low transcript levels at the green fruit stage, highest transcript levels at the pink fruit stage, and lower levels at the blue fruit stage. Expression of one of the genes, pectate lyase, was similar to what was expected from the number of reads, but was high at both the white and pink fruit stages and actually peaked at the white fruit stage rather than the pink fruit stage. Expression of the cysteine protease gene was different from expected. It was quite low at the pink fruit stage, and peaked at the white fruit stage. Messages encoding certain antioxidant enzymes, like glutathione peroxidase, have been shown to increase during ripening of tomato fruits when pink color becomes visible [33]. Also, messages encoding several enzymes involved in anthocyanin biosynthesis, including chalcone synthase and anthocyanidin synthase, are known to rise significantly as color develops in fruits of bilberry or European blueberry (*V. myrtillus*) [34]. Pectate lyases have been shown to play a role in fruit softening during ripening in some plants [35]. Indeed, antisense inhibition of expression of a pectate lyase gene in strawberry indicates that this gene plays an important role in degradation of the primary cell wall and middle lamella in ripening fruit [36].

Figure 7B shows transcript levels across the four stages of fruit ripening (green, white, pink, and blue) for three genes (aspartic proteinase, 1-aminocyclopropane-1-carboxylate oxidase, and flavonoid 3-hydroxylase) whose expression levels appeared to peak at the blue fruit stage based on their number of reads. From real-time PCR, two of the genes (1-aminocyclopropane-1-carboxylate oxidase and flavonoid 3-hydroxylase) showed the expected trends in terms of their gene expression patterns, with highest levels of expression at the blue fruit stage. The gene for aspartic proteinase, however, actually had low levels of expression throughout the different stages of fruit development, but somewhat higher at the white fruit stage than the other stages. It makes sense that the level of transcripts encoding flavonoid 3-hydroxylase, another enzyme involved in anthocyanin biosynthesis, should peak at the blue fruit stage. Finding transcripts encoding 1-aminocyclopropane-1-carboxylate oxidase, an enzyme involved in ethylene biosynthesis, to peak at the pink and blue fruit stages is consistent with findings in strawberry, which have demonstrated increased synthesis of ethylene and ethylene receptors during ripening [37], even though it, like blueberry, is a non-climacteric fruit.

Overall, we found 14 out of 17 (if we include the gene for pectate lyase), or 82%, of the genes examined to have differential expression patterns similar to what would be predicted from their reads alone. In addition, we BLASTed genes identified in *Arabidopsis*, as either being part of the CBF-regulon (cold-response pathway turned on by the transcription factor CBF) or as being cold-response regulatory genes [38,39], against our various 454 sequence assemblies (data not shown). We found homologs to many of these genes among the sequences in our database, such as genes for galactinol synthase, dehydrin cor47, dehydrin erd10, pyruvate decarboxylase, CBF, and ICE (inducer of CBF expression). We also searched our fruit assemblies for enzymes known to be involved in anthocyanin biosynthesis in other plants, and found homologs to all of these genes in our database as well (data not shown). These results suggest that this new database will be very useful for identifying genes that are differentially expressed and play important roles in flower bud development, cold acclimation, chilling unit accumulation, and fruit development.

### Mining database for SSRs

In order to evaluate the usefulness of this database for marker development, the total assembled 454 sequences were mined for SSRs, after removal of sequences shorter than 120 nucleotides. This left 87,071 unique transcript sequences. From these, 15,886 blueberry EST-SSR loci were identified (Table 2). Dinucleotide was the most common repeat motif with a frequency of 50%, followed by tri- (25.9%), tetra-(14.3%), and pentanucleotide (9.8%) repeat motifs. Among the dinucleotide repeats (DNRs), AG/CT repeats (6,034) were the most common in the dataset, accounting for 76% of the total DNRs (7,942) (Table 2). These results are consistent with the frequency of DNRs identified in *Arabidopsis*, rice, soybean, maize, oil palm, coffee, barley, wheat, rubber tree and castor bean [40]. Kantety et al. [41] suggested that the high frequency of AG/CT motifs could be due to the high level of occurrence of the translated amino acid products Glu (GAG), Arg (AGA), Leu (CUC), and Ser (UCU), because their codons carry these motifs. By inspecting the codon usage in 200 ORFs in castor bean, Qiu et al. [40] found a high frequency (24.6% of the total codons analyzed) for these four amino acids, supporting Kantety et al.'s [41] suggestion. CG/GC was the most rare DNR (0.4% of DNRs) found in the blueberry sequences, also in accordance with that reported in other plants [40,41].

**Table 2:** Summary of microsatellite repeats in unique 'Bluecrop' ESTs

Repeats	Count	Percent of Category	Percent of Total
DNR	7942	50.0	9.1

TNR	4120	25.9	4.7

TetraNR	2274	14.3	2.6

PentaNR	1550	9.8	1.8

Total No. of SSRs	15886	-	18.2

**Dinucleotide Repeats**			

CG/GC	31	0.4%	0.2%

TG/GT/AC/CA	530	6.7%	3.3%

AT/TA	1347	17.0%	8.5%

AG/GA/TC/CT	6034	76.0%	38.0%

Total Dinucleotide Repeats	7942		50.0%

**Trinucleotide Repeats**			

AAC/CAA/ACA/GTT/TTG/TGT	177	4.3%	1.1%

AAG/GAA/AGA/CTT/TTC/TCT	1310	31.8%	8.2%

AAT/TAA/ATA/ATT/TTA/TAT	305	7.4%	1.9%

ACC/CAC/CCA/GGT/GTG/TGG	600	14.6%	3.8%

ACG/CGA/GAC/CGT/GTC/TCG	159	3.9%	1.0%

ACT/CTA/TAC/AGT/TAG/GTA	246	6.0%	1.5%

AGC/CAG/GCA/TGC/CTG/GCT	426	10.3%	2.7%

AGG/GGA/GAG/TCC/CTC/CCT	424	10.3%	2.7%

ATC/CAT/TCA/GAT/ATG/TGA	327	7.9%	2.1%

CCG/CGC/GCC/GGC/GCG/CGG	146	3.5%	0.9%

Total Trinucleotide Repeats	4120		25.9%

Among the trinucleotide (TNR) motifs found in blueberry ESTs, AAG/CTT was the most frequently occurring (31.8%), followed by ACC/GGT (14.6%), and AGC/GCT and AGG/CCT (15.0%) (Table 2). Our findings agree with other reports that AAG/CTT is the most prevalent TNR and CCG/CGG is relatively rare in dicotyledonous plants [40,42].

Primers were designed from 7,705 of the SSR-containing sequences. Lack of adequate flanking sequence was the most common reason for not designing primers from the remaining 8,181 SSR-containing sequences. One hundred primer pairs were tested for amplification and polymorphism in a tetraploid *V. corymbosum *mapping population (F1 population resulting from a cross between 'Draper' and 'Jewel') and in an interspecific diploid mapping population (true testcross population resulting from a cross between F1 hybrid #10 [Fla4B (*V. darrowii*) × W85-20 (diploid *V. corymbosum*)] and W85-23 (diploid *V. corymbosum*). Details of results are shown in Additional file 2. In summary, 32 primer pairs failed to generate a product in all accessions while one, VCB-C-13051, only failed in the diploid accessions, not surprising given that ESTs were developed from a tetraploid *V. corymbosum *cultivar, Bluecrop. This actually indicates a very high rate of cross transference into *V. darrowii *and diploid *V. corymbosum*, which agrees with the previous report of Boches et al. [43]. Of the remaining 67 SSR primer pairs that generated a product, 25 were monomorphic in the tetraploid and diploid accessions tested, while 43 resulted in polymorphic products. Six of these 43 were polymorphic only in parents and the two progeny individuals of the tetraploid mapping population (including VCB-C-13051); and 11 were polymorphic only in parents of the diploid mapping population. Therefore, the overall amplification rate was 68% and the polymorphism rate was 43%. Due to the very small number of genotypes assayed, this polymorphism rate is underestimated and is expected to be higher when evaluating a large number of diverse blueberry accessions.

## Conclusions

We have generated a large collection of transcript sequences from the commercial highbush blueberry (*V. corymbosum *L.) using next generation 454 sequencing technology. Transcriptome sequences were obtained from nine different libraries including fruit at four different stages of development, flower buds at four different stages of cold acclimation, and leaves. Over 600,000 reads were assembled into approximately 15,000 contigs and 124,000 singletons, which were annotated and functionally mapped to GO terms. Frequency of the most abundant sequences in each of the libraries was compared across the other libraries to identify genes that are potentially differentially expressed during cold acclimation and fruit development. Real-time PCR confirmed the differential expression patterns of most of the genes that were analyzed. The assembled sequences were also mined for SSRs and over 15,000 blueberry EST-SSR loci were identified. This collection of ESTs should prove to be an important resource for the scientific community particularly for those interested in biological processes such as flower bud development, cold acclimation, chilling unit accumulation/vernalization, flowering, and fruit development, and for those interested in development of molecular markers and genetic linkage maps in blueberry and related species.

From this EST database, we are currently identifying candidate genes for several horticulturally significant traits, such as cold hardiness, chilling requirement, fruit color, etc. based on predicted or real gene expression patterns in blueberry or other plants. We are attempting to map these candidate genes as EST-PCR markers [14] in our mapping populations to determine if they map to the same regions as QTL for these traits. If the genes themselves cannot be mapped due to lack of length polymorphisms, then we are attempting to identify and map SSRs near the genes. This work is being done through a collaboration with Dr. Allan Brown (North Carolina State University), who is heading up an effort to sequence and assemble the whole blueberry genome.

## Methods

### Plant material

Leaves, flower buds, and fruit were collected from multiple plants of the highbush blueberry cultivar Bluecrop grown at the USDA/ARS, Beltsville Agricultural Research Center, Beltsville, MD. 'Bluecrop' was chosen because it is relatively cold hardy and is the "industry standard" of highbush cultivars. Flower buds were collected from field plants during the fall and winter of 2006-2007 with increasing exposure to chilling temperatures, measured as chill units (hours between 0-7°C). Buds were harvested at 0 (9/7/06), 397 (11/30/06), 789 (1/16/07) and 1333 (3/27/07) chill units. Leaf and fruit samples were collected from plants during the 2008 growing season. Fruit was harvested from field plants at four stages of ripening: green (6/12/08), white (6/27/08), pink (7/8/08) and blue or ripe (7/8/08). All tissues were frozen in liquid nitrogen immediately after harvest and stored at -80°C.

### RNA extraction and cDNA preparation

Total RNA was isolated from the leaf and four bud (0, 397, 789, and 1333 chill units) samples by a modification of the method of Chang et al. [44]. Essentially, two grams of each tissue was ground finely in liquid nitrogen and incubated at 65°C in pre-warmed CTAB extraction buffer. Two chloroform:IAA (24:1) extractions were performed, followed by overnight precipitation with LiCl. RNA pellets were resuspended in DEPC water, precipitated again with ethanol and NaOAc, washed, and finally resuspended in 1 ml DEPC water. For isolation of total RNA from the four fruit (green, white, pink, and blue) samples, the same procedure was used with four grams of tissue and the incorporation of a centrifugation step after initial incubation to remove cell debris [45]. Supernatants were transferred to fresh tubes and chloroform:IAA extractions were performed. Additionally, after overnight LiCl precipitation, pellets were washed two to three times with 70% ethanol to clear pigments [46] and resuspended in 500 μl DEPC water. RNA quality was checked on 1% agarose gels stained with ethidium bromide, and concentration was measured with a NanoDrop ND-1000 (NanoDrop Technologies, USA).

The Promega PolyATtract mRNA kit (Promega Corp., USA) was used to isolate mRNA from the nine total RNAs. Poly(A) RNA was ethanol precipitated, and quality and concentration were assessed with the NanoDrop ND-1000. Clontech's SMART cDNA Library Construction kit (Clontech, USA) was used for cDNA synthesis with a protocol adapted by K.V. Donohue (personal communication), and provided by the Genomic Sciences Laboratory, North Carolina State University. The cDNAs were precipitated with ammonium acetate and ethanol, and resuspended in TE. Quality of the cDNAs was assessed on 1% agarose gels stained with ethidium bromide; quantity was determined with the NanoDrop ND-1000.

### Library construction and 454 sequencing

The nine cDNAs (from leaves, flower buds collected at 0, 397, 789, and 1333 chill units, and fruit collected at green, white, pink, and blue fruit stages) were provided to the Genomic Sciences Laboratory, North Carolina State University, for library construction and 454 sequencing. The nine 454 libraries were constructed essentially as described in Poinar et al. [47] and multiplexed. Generally, the cDNAs were sheared by nebulization to give fragments of about 500 bp. The fragmented cDNAs were ligated to adaptor sequences and immobilized on beads. DNA fragments were denatured to generate single-stranded DNA libraries and amplified by emulsion PCR. Sequencing of the libraries was performed using the 454-GS FLX Titanium sequencing platform (454 Life Sciences, Roche Diagnostics, USA). All raw 454 sequence data were deposited in the Sequence Read Archive of the NCBI, accession numbers SRX100856, SRX100859, and SRX100861-SRX100867.

### Sequence assembly

Sequences were analyzed with the GS FLX software v2.0.01.14 package (454 Life Sciences, Roche). Using normalization, correction, and quality-filtering algorithms, weak signals and low quality sequences were removed; read ends were also screened and trimmed for 454 adaptor sequences. Another filtering step masked SMART PCR primer sequences (Clontech) and removed sequences shorter than 50 nucleotides. The remaining 454 sequences were then assembled into unique putative transcripts (contigs and singletons) using the GS De Novo Assembler, an application of the GS FLX software. Default parameters were used with the exception that overlaps were dropped from 40 bases to 30 bases. Reads were assembled in a variety of ways. First, reads from each library were assembled separately. Then, all reads from buds were assembled, all reads from fruit were assembled, and, finally, total reads were assembled.

### Sequence annotation and determination of number of reads

The 454 contigs and singletons from all the various assemblies of the nine libraries were annotated by the Bioinformatics Laboratory at Towson University. The contig and singleton sequences from the assemblies were batch BLASTed to identify the genes expressed in the respective tissues. A domain finding tool, Interproscan [25], was used to help annotate the sequences. Blast2Go and other custom programs, built with the scripting language PERL, were used to create annotated, tab-delimited tables, which included information on taxonomy, gene function, tissue specificity, and GO terms [24]. Contigs with the highest number of reads from each of the libraries were pairwise aligned using BLAST against all the other eight libraries to determine the number of reads with homology from the other libraries. A cut-off e-value of 1E-5 was used. In this way, first, the most highly abundant sequences were identified in each of the libraries. Second, of these, transcripts that were potentially differentially expressed during cold acclimation and during fruit development were identified. The sequences, along with their respective annotations, were stored in a custom-built relational database. The web-based database was built using SQLServer 2008 and ASP.NET (Microsoft, Redmond, WA, USA). Search and browse capabilities were added to allow scientists to access and search the data from the internet. The database can be accessed from: http://bioinformatics.towson.edu/BBGD454.

### Real-time PCR analysis

Total RNA for real-time PCR analysis was isolated from the four bud (0, 397, 789, and 1333 chill units) and four fruit samples (green, white, pink, and blue/ripe) as described above under the "RNA extraction and cDNA preparation" section. RNA quality was checked on 1% agarose gels stained with ethidium bromide, and concentration was measured with a NanoDrop ND-1000 (NanoDrop Technologies, USA). RNA extracts were treated with DNase I, amplification grade (Invitrogen, USA) prior to cDNA synthesis. Complementary DNAs were synthesized using SuperScript III Platinum Two-Step qRT-PCR Kit (Invitrogen).

The genes chosen for qRT-PCR were some that appeared to be differentially expressed during cold acclimation and during fruit development based on their number of reads in the various libraries. In addition, two well-characterized genes, encoding galactinol synthase [9] and a 14 kDa dehydrin [29], already known to be highly induced in blueberry during cold acclimation, were included in the flower bud analyses. The primers for all genes were designed using the Primer Express 3.0 software package (Applied Biosystems, USA). All primer sequences are listed in Table 3. All reactions were run in triplicate on an Applied Biosystems 7500. PCR conditions were 50°C - 2 min, 95°C - 10 min followed by 40 cycles at 95°C - 15 s, 60°C - 1 min. The *Power *SYBR Green PCR Master Mix (Applied Biosystems) was used for the qPCR reactions according to the manufacturer's protocol. Each reaction contained 500 ng of cDNA. All qRT-PCR data were analyzed using the DataAssist V3.0 software package (Applied Biosystems). The maximum CT (cycle threshold) value was set to 40.0 for all analyses. In the past, the blueberry metallothionein gene was used for normalization, as expression of this gene was shown to be constant in cold acclimating buds [48]. Since we were unsure what gene could be used in the fruit samples for normalization, we selected a few candidate genes for testing. These included the high mobility group family protein, a hypothetical protein, and S-adenosyl-1-homocysteine hydrolase. These common genes across reactions were used for global normalization and calculation of relative expression levels by the ΔΔCT method (using DataAssist V3.0). For buds, Bud 0' was set to 1 and for fruit, 'Green' was set to 1.

**Table 3:** Primer sequences for quantitative real-time PCR

Gene name	Primer sequences-forward (F) and reverse (R)	Target Tissue
1-aminocyclopropane-1-carboxylate oxidase	F-TGCAGGTGCCGGGTAGAT R-ATGTCCCTAGCCTCGTTCTACAAC	Fruit

Abscisic stress ripening	F-CCAATTTCACAACCTCGCTCTA R-TGGTGATCGGAAAAGGAAAAA	Bud

Amino acid selective channel protein	F-AATGGAGTATGGGATGGAGAGAGT R-CCCCGAGCATTGCATTCTT	Bud

Anthocyanidin synthase	F-TTCGTGGGCGGCTTTCT R-GAGCGGCTTCAGGATGATCT	Fruit

Arginine decarboxylase	F-CGCTTCGTGCGAGCTAGATA R-TGCAATAGTCTCTCACCACTCGAT	Bud

Aspartic proteinase	F-GAGTGCCATCGGTATAGCACAA R-CACAGGCAGAGCCCCAAA	Fruit

Chalcone synthase	F-AGGCCTTCCAGCCTTTGG R-GGTGGGCGATCCAGAAGAT	Fruit

Cysteine protease-like protein	F-CAACGGTTGAGACCATGGAAT R-TGCAGAGGTCCCCATGCT	Fruit

Dehydrin-14 kDa	F-CGCGGCGATTAGATCGAA R-AGCTAGCGTAGAGGCGGAAA	Bud

Flavonoid 3-hydroxylase	F-GCCGGTGCAACCCACTT R-GGCCGTAGTTGGCGAAAAC	Fruit

Galactinol synthase	F-AAGGGTGTGGTGGGATTGG R-CCAAAGGGTACGCGC CTT	Bud

Glutathione peroxidase	F-TCTAGCATCCTTGACGGTGAAG R-GGCAAGCCAATCCGAGAAG	Fruit

High mobility group family protein	F-CGTCATGAAAGGAGGTAAATCCA R-TTTCGCAGTTTTCTTCACCGATA	Bud/Fruit housekeeping gene

Hypothetical protein	F-CCTGCTGCATCAAGCTGTTG R-TGGCTGGAAGCACTCACTGT	Bud/Fruit housekeeping gene

Lipid transfer protein	F-CCAAGGTGAAATGAGCAAGGA R-GCTTCATCCCGCGACATG	Bud

Lipoxygenase	F-CCAGCTGGATGTGGCAACTT R-ACGCCTGCGTCATTGGA	Bud

Metallothionein	F-ACCCTGACATGAGCTTCTCG R-ACCCAAATCTCTGCTTGCTG	Bud housekeeping gene

Pectate lyase	F-GGTTGCCGGTCCCACAA R-GAGCATCAATGCGTCAAGGA	Fruit

Pointed first leaf	F-CGTTTTGCCAACATCGTTTG R-GCTCACCAGCCCTCTTGTTC	Bud

Pyrophosphate-dependent phosphofructokinase	F-GCCCTTAACAACCGCTACATCA R-TCTGCTACTGGGCCAACAAAT	Bud

S-adenosyl-1-homocysteine hydrolase	F-CACCGGATCGCTTCACATG R-CGGTGAGGGTTTCGATAAGG	Fruit housekeeping gene

### SSR mining

Sequences shorter than 120 nt were removed from the total assembled 454 sequences, leaving 87,071 unique 454 transcript sequences, which were analyzed using the online SSR tool (SSR Server) available at the Genome Database for *Vaccinium *(GDV, http://www.vaccinium.org). SSR Server identifies simple sequence repeats using user-specified motif parameters and generates an EXCEL file containing the identified SSRs and coordinates in the sequence, primers generated from Primer3 [49], and Open Reading Frame coordinates using getORF http://emboss.sourceforge.net/apps/cvs/emboss/apps/getorf.html. SSRs recorded for the final dataset included dinucleotide repeats (DNR) with at least 5 repeats, trinucleotide repeats (TNR) with at least 4 repeats, tetramers with at least 3 repeats, and pentamers with at least 3 repeats. Of these SSR-containing sequences, those that had a GC content between 40 and 60% and a minimum of 20 bases of sequence on either side of the repeat motif were selected as optimal candidates for primer development.

M13-tailed forward primers, as described by Schuelke et al. [50], and standard reverse primers were obtained for 100 SSR-containing sequences. These primers were tested for amplification and polymorphism in a tetraploid and a diploid mapping population of blueberry. Individuals tested in the highbush tetraploid (*V. corymbosum*) mapping population were parents 'Draper' and 'Jewel' and two progeny individuals, BB 05-61-1 and BB 05-61-2. Individuals tested in the interspecific diploid testcross mapping population included the parents #10 [Fla4B (*V. darrowii*) × W85-20 (diploid *V. corymbosum*)] and W85-23 (another diploid *V. corymbosum*), along with the original parents of #10, Fla4B and W85-20. PCR reactions (15 μL total volume) contained 1X reaction buffer, 2 mM MgCl_2_, 0.2 mM dNTPs, 0.5 μM of the fluorescent M13 primer, 0.12 μM forward primer, 0.50 μM reverse primer, 0.075 units of GoTaq^® ^DNA Polymerase (Promega, USA), and 4.5 ng genomic DNA. The touchdown PCR temperature profile used for amplification followed: one cycle of 94°C for 3 min; 10 cycles of 94°C for 40 s, 65°C for 45 s (-1.0°C per cycle), and 72°C for 45 s; 20 cycles of 94°C for 40 s, 52°C for 45 s, and 72°C for 45 s; eight cycles of 94°C for 40 s, 53°C for 45 s, and 72°C for 45 s, one cycle at 72°C for 30 min. Once PCR success was assessed by 2% agarose gel electrophoresis, PCR products generated from up to four primer pairs were pooled and separated by capillary electrophoresis using the Beckman CEQ 8000 genetic analyzer (Beckman Coulter, USA) for all eight samples.

## Abbreviations

ASR: Abscisic Stress Ripening; bp: Base pairs; CBF: C-Repeat Binding Factor; DNR: Dinucleotide Repeat; EST: Expressed Sequence Tag; GO: Gene Ontology; ICE: Inducer of CBF; LT_50_: Lethal Temperature resulting in 50% death; NCBI: National Center for Biotechnology Information; Nr: Non-redundant; Nt: Nucleotides; ORF: Open Reading Frame; qRT-PCR: Quantitative Real-Time PCR; QTL: Quantitative Trait Loci; SSR: Simple Sequence Repeat; TNR: Trinucleotide Repeat.

## Authors' contributions

LJR designed and directed the study, contributed to the collection of tissue samples, participated in the analysis of data including selecting genes for real-time PCR, and drafted the manuscript. NA and OD performed the bioinformatic analyses and contributed to writing relevant sections of the manuscript. ELO contributed to the tissue sample collection, optimized and wrote up the RNA extraction protocols, performed the RNA extractions, and prepared the cDNAs for construction of 454-libraries and for use in real-time PCR. JJP designed the real-time PCR primers, performed the real-time PCR experiments, and contributed to writing relevant sections of the manuscript. DM mined the 454 sequences for SSRs and designed SSR primers. NVB tested the SSR primers on two blueberry mapping populations and contributed to writing relevant sections of the manuscript. All authors read and approved the final manuscript.

## Supplementary Material

Additional file 1 **Most highly abundant sequences from each of the libraries along with their number of reads/frequency of reads and predicted expression patterns across the bud time points and the fruit development stages**. Approximately 30 most highly abundant sequences from each of the libraries along with their number of reads/frequency of reads and predicted expression patterns across the bud time points (0, 397, 789, and 1333 chill hours) and fruit development stages (green, white, pink, and blue/ripe). Click here for file

Additional file 2 **Summary of amplification and polymorphism results from screening 100 EST-SSRs on parents of tetraploid and diploid mapping populations of blueberry**. Result summary of amplification and polymorphism of 100 EST-SSRs in parents of a tetraploid mapping population 'Draper' and 'Jewel' and two progeny individuals, BB 05-61-1 and BB 05-61-2; and in a diploid testcross mapping population between #10 [Fla4B (*V. darrowii*) × W85-20 (*V. corymbosum*)] and W85-23. Fla4B and W85-20 were also included. Prefix VCB is in the SSR name and precedes the EST name and stands for *Vaccinium corymbosum *'Bluecrop', the source of the ESTs. Polymorphic alleles are highlighted. Y indicates in ORF while N indicates absence from ORF. Click here for file
