# Supplementary material for: Generation and analysis of blueberry transcriptome sequences from leaves, developing fruit, and flower buds from cold acclimation through deacclimation
Source: BMC Plant Biol. 2012 Apr 2;12:46. doi: 10.1186/1471-2229-12-46 (PMC3378433; doi:10.1186/1471-2229-12-46)
Supplement: Additional file 2 — Summary of amplification and polymorphism results from screening 100 EST-SSRs on parents of tetraploid and diploid mapping populations of blueberry. Result summary of amplification and polymorphism of 100 EST-SSRs in parents of a tetraploid mapping population 'Draper' and 'Jewel' and two progeny individuals, BB 05-61-1 and BB 05-61-2; and in a diploid testcross mapping population between #10 [Fla4B (V. darrowii) × W85-20 (V. corymbosum)] and W85-23. Fla4B and W85-20 were also included. Prefix VCB is in the SSR name and precedes the EST name and stands for Vaccinium corymbosum 'Bluecrop', the source of the ESTs. Polymorphic alleles are highlighted. Y indicates in ORF while N indicates absence from ORF. [file 1471-2229-12-46-S2.DOCX]

Additional file 2. Result summary of amplification and polymorphism of 100 EST-SSRs in parents of a tetraploid mapping population ‘Draper’ and ‘Jewel’ and two progeny individuals, BB 05-61-1 and BB 05-61-2; and in a diploid testcross mapping population between #10 [Fla4B (*V. darrowii*) x W85-20 (*V. corymbosum*)] and W85-23. Fla4B and W85-20 were also included. Prefix VCB is in the SSR name and precedes the EST name and stands for *Vaccinium corymbosum* ‘Bluecrop’, the source of the ESTs. Polymorphic alleles are highlighted. Y indicates in ORF while N indicates absence from ORF.

| **SSR** | **Motif** | **ORF** | **Primers (5'-3')** | **ExpectedSize** | **Swissprot or TrEMBL homolog** | **E-value** | **Alleles in** | |
| --- | --- | --- | --- | --- | --- | --- | --- | --- |
|  |  |  |  |  |  |  | ***V. corymbosum* (4x)** | ***V. darrowii*/*V. corymbosum* (2x)** |
| VCB-C-02051 | (tc)5 | Y | F-GGAAGTCCTTTTCCAGCTCTCT | 100 | *Vitis vinifera* whole genome shotgun sequence of line PN40024, scaffold_23.assembly12x | 5E-10 | 119 | 119 |
|  |  |  | R-ATCGGAAGGAAAGGAAAGAGTC |  |  |  |  |  |
| VCB-C-02476 | (ataa)3 | N | F-AATCGACTTCTGCTTCTCTTGC | 100 | *Bos taurus* proteasomal ubiquitin receptor ADRM1 | 3E-09 | 118 | 118 |
|  |  |  | R-GGAGATAGTTGAAGCCACTGCT |  |  |  |  |  |
| VCB-C-04624 | (ctc)5 |  | F-GTTCATCCCAATGCAGAAGAAG | 100 | *Ricinus communis* putative uncharacterized protein | 7E-24 | 114, 117, 120 | 104, 114, 117 |
|  |  |  | R-CCTCTTGTGGGTTAGGGTTTCT |  |  |  |  |  |
| VCB-C-06772 | (ttct)3 | Y | F-GTTCTTTGGAGTTGTTCCAACC | 100 | *Ricinus communis* putative uncharacterized protein | 1E-53 | 118 | 118 |
|  |  |  | R-AAGGAGAACTGCTTCTCGAATG |  |  |  |  |  |
| VCB-C-11525 | (ac)5 | N | F-AACTGAAGAACAAACCATTGCC | 100 | *Homo sapiens* monoglyceride lipase | 0.0000002 | 117 | 117 |
|  |  |  | R-GCAACTATTTGTGTGGGTTGAA |  |  |  |  |  |
| VCB-C-11818 | (tct)6 | N | F-GGTTCTCTTGTTTGAATAGGCG | 100 | *Vitis vinifera* whole genome shotgun sequence of line PN40024, scaffold_116.assembly12x | 2E-30 | 117, 119, 125, 132 | 119, 122, 132, 141, 155 |
|  |  |  | R-GAAACAAACGGAGCCTAAAAGA |  |  |  |  |  |
| VCB-BH-1BQCRI | (ct)8 | N | F-GAGGAGAAACACAAGCACCAA | 100 |  |  | 119, 121, 123, 125, 127 | 121, 123, 125 |
|  |  |  | R-GAGAAGGCAAGCAGAGAGACTG |  |  |  |  |  |
| VCB-BH-1C6ZD8 | (tct)5 | N | F-TTGTGAGTTGACTCATGCTTCC | 100 |  |  | 116 | 116 |
|  |  |  | R-CCTTGGAATTAGAATTCGGACA |  |  |  |  |  |
| VCB-BH-1CC8GL | (aaaca)4 | Y | F-AAAATTAAAACGGAACCGGG | 100 |  |  | NA | NA |
|  |  |  | R-CTTTTCTGCCTGCCATTGTACT |  |  |  |  |  |
| VCB-BH-1CFWRP | (tgg)4 | Y | F-GCTCGACAAGGTAGGTGGTG | 100 |  |  | NA | NA |
|  |  |  | R-TTCCTCTTTCTTTGTCTTCTGC |  |  |  |  |  |
| VCB-BH-1CL12I | (ctt)5 | Y | F-GTTCTGTTTCTTTTGGGGGTTT | 100 |  |  | NA | NA |
|  |  |  | R-CTACGGCACTTTCCCCATAAT |  |  |  |  |  |
| VCB-C-04025 | (ct)14 | N | F-CTCAGCCATATGAAGTGTAGCG | 104 | *Arabidopsis thaliana* transcription factor bHLH48 | 9E-66 | NA | NA |
|  |  |  | R-CAAACCCAAACCACACAATACA |  |  |  |  |  |
| VCB-C-09425 | (ctc)5 | N | F-CCTTAAAAATCTCCACCGTCAC | 125 | *Glycine max* putative uncharacterized protein | 6E-24 | 140, 143 | 140 |
|  |  |  | R-CGTCGATGTAAACTCGTCTCTG |  |  |  |  |  |
| VCB-C-10090 | (ga)5 | N | F-GTCAACACCATTGGACCCTTAT | 125 | *Populus trichocarpa* predicted protein | 3E-41 | 144 | 144 |
|  |  |  | R-CGACTCATTTCACACCTTTCAC |  |  |  |  |  |
| VCB-C-13051 | (tc)5 | N | F-CTCTTTCTTTTTCTTTCACCGTCTG | 125 |  |  | 143, 153 | NA |
|  |  |  | R-TTTCCTTCTGGCTCTTAGATCG |  |  |  |  |  |
| VCB-BH-1A71SQ | (gt)5 | Y | F-AAGAATGCAACACACAACATGG | 125 |  |  | 139, 143, 147, 149 | 137, 139, 143 |
|  |  |  | R-GGGGGAGTCTTTTGAAACTCTT |  |  |  |  |  |
| VCB-BH-1BLNKR | (ct)8 | Y | F-ATATACGCACACAAAAATCCCC | 125 |  |  | 136, 141, 143, 151, 153 | 141, 143 |
|  |  |  | R-GCCCTAAGTTGGAATTGGTTTC |  |  |  |  |  |
| VCB-BH-1CCTBS | (tc)6 | N | F-CAGCAGCAGTATCAGCTTGACT | 125 | *Paulownia kawakamii* superoxide dismutase [Cu-Zn] | 8E-45 | NA | NA |
|  |  |  | R-ACTGCTGTTAAGAACGACGACA |  |  |  |  |  |
| VCB-BH-1CS1IE | (tc)6 | N | F-CAGCAGCAGTATCAGCTTGACT | 125 | *Ananas comosus* superoxide dismutase [Cu-Zn] | 3E-35 | NA | NA |
|  |  |  | R-ACTGCTGTTAAGAACGACGACA |  |  |  |  |  |
| VCB-BH-1D6JIL | (ac)9 | Y | F-GCCATGCATTCTGAAGGAAA | 125 |  |  | NA | NA |
|  |  |  | R-GTTTCTCTAGGCCTGAGGAGGT |  |  |  |  |  |
| VCB-BH-2FTPY0 | (at)5 | Y | F-TCTCTAATTCTAGAGGCCGAGG | 125 |  |  | NA | NA |
|  |  |  | R-TTGTATTTGTGGACCGGTAGTG |  |  |  |  |  |
| VCB-BH-2G2M6V | (tttc)4 | N | F-CGCGTGTCCTATTAGTGGTCTT | 125 |  |  | NA | NA |
|  |  |  | R-ATAGCTCTCTCCAACCTTTCCC |  |  |  |  |  |
| VCB-QO-1A7ERL | (taga)3 | Y | F-ATGCATACCAGTACTGCTCCCT | 125 |  |  | 137, 141 | 137, 141 |
|  |  |  | R-AGGTGAAGGCTAAAACGATGTC |  |  |  |  |  |
| VCB-C-02477 | (aag)4 | N | F-GGGATTTTAAACAGCATACCCA | 150 | *Arabidopsis thaliana* thioredoxin-like protein CXXS1 | 2E-23 | 321, 331, 338, 343, 367 | 331, 338, 341, 345, 367 |
|  |  |  | R-GCTTGGGATAAATAGGATTCCC |  |  |  |  |  |
| VCB-C-03545 | (tgac)3 | Y | F-TGAGTATCATATGACTTGGGCG | 150 | *Ricinus communis* multidrug resistance protein, putative | 0 | 300, 303, 320, 330, 343, 367 | 330, 326, 343, 349 |
|  |  |  | R-GAGCGAGTAGGAGAGTTTTCCA |  |  |  |  |  |
| VCB-C-04055 | (gaa)6 | N | F-ACTCCGATTCCGACGACTCT | 150 | *Arabidopsis thaliana* uncharacterized protein At4g19900 | 8E-84 | 168, 184, 186, 214 | 214 |
|  |  |  | R-CTCCACTCACGTGATCGAAGTA |  |  |  |  |  |
| VCB-C-05571 | (tcc)5 | N | F-CTAGAGTGCAATCAAGGCCAAT | 150 | *Ricinus communis* putative uncharacterized protein | 1E-24 | 169 | 166, 169 |
|  |  |  | R-CGGACCTTGAAGACCTAGCTTA |  |  |  |  |  |
| VCB-C-13245 | (ccat)3 | N | F-CTTGAGGCCCTTCTTGTCAC | 150 | *Glycine max* 4-coumarate--CoA ligase 2 | 3E-40 | NA | NA |
|  |  |  | R-GGCGATGTATTCTTTAACAGCC |  |  |  |  |  |
| VCB-BH-1DC9CL | (tttc)3 | Y | F-GATGAAAGCGAGAGAATTGGTC | 150 |  |  | 172 | 172 |
|  |  |  | R-ATAGCTCTCTCCAACCTTTCCC |  |  |  |  |  |
| VCB-BH-1DVIYK | (ct)6 | Y | F-CTTCAAACTCCTCTCTCCTCCA | 150 |  |  | 148, 168, 171, 181 | 148, 168, 183, 192 |
|  |  |  | R-ACTTCTCGACGTGCCTTACTTC |  |  |  |  |  |
| VCB-BH-1E0G7J | (ct)7 | N | F-GGGAATTTCTTGTTGACTCCAG | 150 |  |  | NA | NA |
|  |  |  | R-TGGTTCGACAGTTGGTTGTAAG |  |  |  |  |  |
| VCB-BH-2FXQ1U | (ttta)4 | Y | F-TGAGGTGAATTTGGAGGAGTTT | 150 |  |  | 158, 168, 174 | 158, 168 |
|  |  |  | R-CCACTAGTGTTGAAGACGGGAT |  |  |  |  |  |
| VCB-BH-2G6V5E | (caga)3 | Y | F-GAACATAGGACAGACGGAGGAC | 150 |  |  | 171 | 171 |
|  |  |  | R-ACGTCGAAGCACTCAGATGTT |  |  |  |  |  |
| VCB-BH-2H78QI | (tg)5 | Y | F-AAGCTTTTTGTGTACCCATGCT | 150 | *Populus trichocarpa* predicted protein | 0.00000003 | 169, 171 | 171 |
|  |  |  | R-ACTTTCGAAACAGTACGGCATT |  |  |  |  |  |
| VCB-C-05700 | (tc)9 | N | F-CAACAGCTCATAGAGAAAGGGC | 175 | *Mus musculus* trimethylguanosine synthase | 9E-29 | 194, 196, 198, 202, 207, 209, 225 | 196, 207, 209, 211, 215 |
|  |  |  | R-AGCATTCTCTTGACTAGACGGC |  |  |  |  |  |
| VCB-C-11552 | (ttca)3 | N | F-TGGAAAACCACGGTTAATAAGG | 175 | *Ricinus communis* zinc finger protein, putative | 2E-12 | 193 | 193 |
|  |  |  | R-ATGGTGCATACCCTTAATTTCG |  |  |  |  |  |
| VCB-C-11858 | (gag)5 | Y | F-AATCAAGCCCAGAGAGAAATGA | 175 | *Vitis vinifera* whole genome shotgun sequence of line PN40024, scaffold_1.assembly12x | 3E-71 | 195 | 195, 198 |
|  |  |  | R-CTCCTCCTCTACCCCTCAGATT |  |  |  |  |  |
| VCB-C-13063 | (ag)6 | N | F-GACAACGAATACGGTGACAAAA | 175 | *Ricinus communis* putative uncharacterized protein | 1E-33 | 191 | 191 |
|  |  |  | R-ACGCCCAAGTAGAAAAATCGTA |  |  |  |  |  |
| VCB-C-14729 | (ga)5 | Y | F-AGACCCAATGACCTGTCCTAGA | 175 | *Populus trichocarpa* predicted protein | 1E-87 | 194 | 194 |
|  |  |  | R-TAACATCCCTCTGGGAATATGG |  |  |  |  |  |
| VCB-C-14758 | (tc)10 | N | F-AGAGATGCTGGTGGATCTTGTT | 175 | *Vitis vinifera* whole genome shotgun sequence of line PN40024, scaffold_62.assembly12x | 3E-79 | 193, 197 | 191, 193 |
|  |  |  | R-TGATCATCAGCTTCCGTTTCA |  |  |  |  |  |
| VCB-BH-1A51SM | (ga)6 | N | F-TTCTGAGGAATTGGGAAACAGT | 175 | *Solanum bulbocastanum* dopamine-responsive protein, putative | 1E-09 | 195 | 195 |
|  |  |  | R-GGAAAGCTCTCCTCACGTTTT |  |  |  |  |  |
| VCB-BH-1BFB77 | (ta)6 | N | F-ATATCGCGAGAAGCAGTGGTAT | 175 |  |  | NA | NA |
|  |  |  | R-CTCGACATTTAAGACCTCCGTC |  |  |  |  |  |
| VCB-BH-1BQQY7 | (ga)6 | N | F-AAGAAGTGGACCAATTTAACGG | 175 | *Nicotiana tabacum* 60S ribosomal protein L23 | 4E-17 | NA | NA |
|  |  |  | R-ATGATGTAGAGGTTCTTGGCCC |  |  |  |  |  |
| VCB-BH-1BVTGV | (cga)4 | N | F-ATATCGCGAGAAGCAGTGGTAT | 175 |  |  | NA | NA |
|  |  |  | R-TCAGAAGTTAGGTTGGCGTCTT |  |  |  |  |  |
| VCB-BH-2H94XO | (cag)5 | N | F-AGCAGCAGGAGACTTAATCGAC | 175 |  |  | NA | NA |
|  |  |  | R-AGAAGCGAAGAACCGAAGAAG |  |  |  |  |  |
| VCB-C-00847 | (ag)8 | Y | F-AAGCTTAGTTGCCAGTGTGTGA | 200 |  |  | NA | NA |
|  |  |  | R-CGGGCTTCTTTGTACGAATACT |  |  |  |  |  |
| VCB-C-01394 | (ga)5 | N | F-CTTGATCTCTCAGGCACCTCTT | 200 | *Catharanthus roseus* secologanin synthase | 9E-141 | 219 | 219 |
|  |  |  | R-ACAGCCCCACATGTAGGATAAG |  |  |  |  |  |
| VCB-C-01424 | (tcaa)3 | N | F-CCATAGAGTGCCTACCCCAGTA | 200 | *Arabidopsis thaliana* auxin-responsive protein IAA27 | 4E-80 | 220, 222 | 220, 233 |
|  |  |  | R-ATTGAAGAAGCCTCTGACAAGC |  |  |  |  |  |
| VCB-C-03218 | (cata)3 | N | F-TGTTAAACGCTAACCGTTCCTT | 200 | *Arabidopsis thaliana* chaperone protein dnaJ 8, chloroplastic | 6E-25 | 220, 225 | 220, 225 |
|  |  |  | R-CACCGTTAATAAACCCAGCAGT |  |  |  |  |  |
| VCB-C-03932 | (tgttt)3 | Y | F-GACGATGGATTTGAGGAGAAAG | 200 | *Arabidopsis thaliana* DEAD-box ATP-dependent RNA helicase 36 | 2E-45 | 219 | 219 |
|  |  |  | R-TCACACAAGCACACAATTACCA |  |  |  |  |  |
| VCB-C-07227 | (ggc)5 | N | F-GAATTGATCAGCTCTTGCTGTG | 200 | *Arabidopsis thaliana* probable thiol methyltransferase 2 | 8E-69 | 215, 222 | 215, 227, 234 |
|  |  |  | R-GTCTCCGGTTATAGTCGTCCAC |  |  |  |  |  |
| VCB-C-08247 | (tgc)4 | Y | F-TGTTCCTCTAGCTGGTTGTTGA | 200 | *Arabidopsis thaliana* auxin response factor 8 | 4E-32 | 220 | 220 |
|  |  |  | R-GCCGGTTTACAGAATGTAGGAG |  |  |  |  |  |
| VCB-BH-2F18OJ | (ttc)5 | Y | F-GCTCCAAGCTTTTATGTATCCG | 200 |  |  | NA | NA |
|  |  |  | R-GTGACTGGGAATGATTAGAAAGAAG |  |  |  |  |  |
| VCB-BH-2JO3U8 | (ga)5 | N | F-ATATCGCGAGAAGCAGTGGTAT | 200 |  |  | NA | NA |
|  |  |  | R-CTCTTCGAACCATTTCTCCACT |  |  |  |  |  |
| VCB-QO-1C7KOJ | (agc)5 | Y | F-TATCATGACCTGCTTTGTTTGG | 200 | *Glycine max* cytochrome P450 78A3 | 2E-12 | 220, 223 | 220, 223 |
|  |  |  | R-ACATGAGATTCATGCTCCCAAT |  |  |  |  |  |
| VCB-QO-1D9EOB | (ttca)4 | N | F-ACTAAGAAGAACCGAAGGACGG | 200 | *Urechis caupo* histone H3 | 2E-22 | NA | NA |
|  |  |  | R-GTATAGATGACGCGTTTAGGGC |  |  |  |  |  |
| VCB-C-02780 | (aatg)3 | N | F-CTTATCGCAGTAGTGGACATCG | 225 | *Dictyostelium discoideum*  putative phosphatidylglycerol/phosphatidylinositol transfer protein DDB_G0282179 | 1E-15 | 240, 244 | 240, 244 |
|  |  |  | R-CTTCTTGTGTTGGTCAACGGT |  |  |  |  |  |
| VCB-C-03874 | (ga)14 | N | F-CGTCAGTAGGCAAAGTTGAGTG | 225 | *Homo sapiens* protoheme IX farnesyltransferase, mitochondrial | 2E-45 | NA | NA |
|  |  |  | R-GGAAGCTTTCTTCTGAAAACTGG |  |  |  |  |  |
| VCB-C-05127 | (ga)5 | Y | F-CAAAATGGTGAGTTCCTTGTGA | 225 |  |  | 237, 247 | 237, 247 |
|  |  |  | R-GTCAATCGAGGTCAAGAAAACC |  |  |  |  |  |
| VCB-C-06984 | (ct)5 | N | F-TAAACTCGCTCCTACGATGGTT | 225 | *Vitis vinifera* whole genome shotgun sequence of line PN40024, scaffold_11.assembly12x | 5E-120 | NA | NA |
|  |  |  | R-ATTGATGGAGCAGGTAAGGAAA |  |  |  |  |  |
| VCB-C-07274 | (aga)4 | N | F-GGGAGTATTGGTACCAGAGCAG | 225 | *Xenopus tropicalis* nuclear factor related to kappa-B-binding protein | 9E-15 | 243 | 243 |
|  |  |  | R-ATGAAGTCCCATCATCCTCAAA |  |  |  |  |  |
| VCB-C-07323 | (gat)5 | N | F-GCTGGTCTTAAGAGCTGGAAAA | 225 | *Homo sapiens* ribosome biogenesis protein BMS1 homolog | 2E-21 | 245 | 245 |
|  |  |  | R-CAATTTTAGTGGCATCCTCCTC |  |  |  |  |  |
| VCB-C-08049 | (ct)5 | Y | F-CACAGGGAAGTTCTTTCCAAAC | 225 | *Oryza sativa* subsp. *japonica* Squamosa promoter-binding-like protein 14 | 0.0000005 | 244 | 244 |
|  |  |  | R-GAAGTGATTACCCGGAGACTTG |  |  |  |  |  |
| VCB-C-09527 | (tgtt)3 | N | F-TATGCAGCTTGGGGTTCTTAAT | 225 | *Arabidopsis thaliana* SNF1-related protein kinase catalytic subunit alpha KIN10 | 2E-114 | 244, 247, 250, 254 | 247, 250, 257, 266 |
|  |  |  | R-CAACCTAGAAAAATGCGCCTAC |  |  |  |  |  |
| VCB-C-12885 | (ctg)4 | Y | F-CTGCTGCCTAACCTCATATTCC | 225 | *Arabidopsis thaliana* BEL1-like homeodomain protein 8 | 7E-12 | 245, 248 | 245 |
|  |  |  | R-TATGATGACCAGCCCACAGTAG |  |  |  |  |  |
| VCB-C-14488 | (tc)9 | N | F-TTTGAGAATTTATCCACCCGAG | 225 | *Solanum tuberosum* ribulose-phosphate 3-epimerase, chloroplastic | 4E-98 | NA | NA |
|  |  |  | R-AACGCCTAACCCACAATTCTAA |  |  |  |  |  |
| VCB-BH-1BM03L | (ta)6 | Y | F-CACCACCAGAGCTAGTTGATCTC | 225 |  |  | 202, 204, 208, 237, 243, 245, 247 | 202, 204, 243, 245 |
|  |  |  | R-TGAATTTGATATGAAGGGGGAC |  |  |  |  |  |
| VCB-C-01748 | (aacc)3 | Y | F-AAACTAGTTGCCACCGGAGTAA | 250 | *Vitis vinifera* putative uncharacterized protein | 6E-65 | 270 | 270 |
|  |  |  | R-CCAAGAAGTGGTCCTTGGTAAG |  |  |  |  |  |
| VCB-C-03432 | (ga)5 | N | F-GCAACCAAAACCTGTAACACAA | 250 | *Populus trichocarpa* predicted protein | 2E-26 | 269 | 269 |
|  |  |  | R-TCCTAACCAAAGCAGAGAAAGC |  |  |  |  |  |
| VCB-C-06829 | (cac)5 | Y | F-CACAACTTGACTGAGAACCAGG | 250 | *Arabidopsis thaliana* AP2-like ethylene-responsive transcription factor ANT | 3E-14 | 269, 272 | 263, 265, 269 |
|  |  |  | R-ACCACAACCCCTACTACAATGG |  |  |  |  |  |
| VCB-C-07210 | (att)4 | N | F-CATTTTGTGGCACTGTCATCTT | 250 | *Vitis vinifera* whole genome shotgun sequence of line PN40024, scaffold_21.assembly12x | 2E-15 | 269, 271 | 269, 271 |
|  |  |  | R-GTTGCTGCTCGGTTTCTTACTT |  |  |  |  |  |
| VCB-C-07361 | (gca)4 | Y | F-AGTGTTTGAACAGGTGGTTGTG | 250 | *Populus trichocarpa* predicted protein | 6E-49 | 269, 275 | 264, 273, 275 |
|  |  |  | R-GCAATGAGTGCAAATGGAGTAA |  |  |  |  |  |
| VCB-C-08295 | (tga)4 | N | F-CAGCTGCTATCTGGTTCACATC | 250 | *Bombyx mori* partner of Y14 and mago | 1E-09 | 268, 270, 273 | 268, 270 |
|  |  |  | R-AACTCTGCAGTCTGCTCACAAC |  |  |  |  |  |
| VCB-C-09326 | (ct)5 | N | F-ATTTCCTCCAAACAGTTCCAGA | 250 | *Poncirus trifoliata* Squamosa promoter-binding protein | 8E-12 | 271 | 271 |
|  |  |  | R-CTCCACATTGTTACCATGCAGT |  |  |  |  |  |
| VCB-C-09892 | (gaa)4 | Y | F-AAGAAGAAGGTGAAGATGGCAG | 250 | Arabidopsis thaliana (Mouse-ear cress)NEP1-interacting protein 1 | 7E-63 | 271, 274 | 271, 274, 277 |
|  |  |  | R-TACCTTCAACGAGACCCAATCT |  |  |  |  |  |
| VCB-C-10992 | (ct)11 | N | F-TAATTGGAAGAGTTCGTGAGGG | 250 | Dictyostelium discoideum (Slime mold)Luc7-like protein | 1E-16 | 263, 267, 269, 273 | 261, 263, 269 |
|  |  |  | R-GTGAAGGCCTGAAGTCTCTGAA |  |  |  |  |  |
| VCB-C-13697 | (ttc)4 | N | F-TTGTCGACGATAATTGTCCAAG | 250 | Synechocystis sp. (strain ATCC 27184 / PCC 6803 / N-1)Uncharacterized protein sll1770 | 2E-23 | 270 | 270, 274 |
|  |  |  | R-ATAAGCCAGTGAAATTGGCAGT |  |  |  |  |  |
| VCB-BH-1APTTM | (tat)4 | N | F-TACCGGTAATTGATCTCGAAGG | 250 | Vitis vinifera (Grape)Whole genome shotgun sequence of line PN40024, scaffold_5.assembly12x | 3E-10 | 138, 264, 267 | 138, 171, 264, 267 |
|  |  |  | R-ATCGAAAACCTCTATCCACACG |  |  |  |  |  |
| VCB-C-01452 | (ttc)14 | Y | F-TCTTTGGTATTTCGAGGGAAGA | 275 |  |  | NA | NA |
|  |  |  | R-CCGGTATAATGGAGAGGGGTAT |  |  |  |  |  |
| VCB-C-02895 | (ag)8 | N | F-ACTCTCCAATGTATCCGTGCTT | 275 | Arabidopsis thaliana (Mouse-ear cress)40S ribosomal protein S15a-1 | 9E-68 | NA | NA |
|  |  |  | R-GATTTCAAGTTTCAGCCTCGC |  |  |  |  |  |
| VCB-C-03938 | (ag)7 | N | F-CCTCAGATAACTGAAACCCGTC | 275 | Arabidopsis thaliana (Mouse-ear cress)Metal tolerance protein 11 | 1E-38 | 294, 296 | 294, 296 |
|  |  |  | R-CCTCTCTATTTTCGGTTTCCCT |  |  |  |  |  |
| VCB-C-05353 | (tct)4 | N | F-CGGGGAAAACAATAAAGATCAG | 275 | Vitis vinifera (Grape)Whole genome shotgun sequence of line PN40024, scaffold_5.assembly12x | 2E-91 | 292, 294 | 294 |
|  |  |  | R-CTGGATGTTGATGAGATGGCTA |  |  |  |  |  |
| VCB-C-06609 | (ct)5 | Y | F-AACAGCGGGATTGTACTTCCTA | 275 | Dictyostelium discoideum (Slime mold)DDT domain-containing protein DDB_G0282237 | 1E-11 | NA | NA |
|  |  |  | R-AACGTGATTGGAGGATCTTCAT |  |  |  |  |  |
| VCB-C-07634 | (tggct)3 | N | F-CTTTCTGGCTATGGCTTTCACT | 275 | Arabidopsis thaliana (Mouse-ear cress)ALA-interacting subunit 3 | 4E-55 | NA | NA |
|  |  |  | R-CCTACAGATGCCACAAATCAAA |  |  |  |  |  |
| VCB-C-10178 | (aagc)3 | Y | F-CCGGCTTTGAGTTACAGAGAAT | 275 | Camellia sinensis (Tea)Retinoblastoma-related protein | 2E-150 | 386 | 386 |
|  |  |  | R-GGAAGAAACATCACTTTCCGTC |  |  |  |  |  |
| VCB-C-10576 | (ct)5 | N | F-GTTTGATAAGCCAATCGAGGAC | 275 | Rattus norvegicus (Rat)ADP-ribosylation factor-like protein 8B | 2E-63 | NA | NA |
|  |  |  | R-GGTCTCCGCCATTGTATATGTT |  |  |  |  |  |
| VCB-C-12765 | (ag)14 | N | F-AGACTTGGATTGATCGGTTGTT | 275 | Populus trichocarpa (Western balsam poplar) (Populus balsamifera subsp. trichocarpa)Predicted protein | 2E-24 | NA | NA |
|  |  |  | R-TAGCTGGAGGATTCTCTCTTGG |  |  |  |  |  |
| VCB-C-13779 | (tc)5 | N | F-GGCTTGCAACATACTCTACAAGG | 275 | Vitis vinifera (Grape)Whole genome shotgun sequence of line PN40024, scaffold_11.assembly12x | 6E-33 | 276, 281, 290, 292 | 276, 292 |
|  |  |  | R-ACTGCTTGTTGATTCCTGACCT |  |  |  |  |  |
| VCB-C-13880 | (cat)9 | N | F-TAAAATAGTTCAATCCACCCCG | 275 | Populus trichocarpa (Western balsam poplar) (Populus balsamifera subsp. trichocarpa)Predicted protein | 1E-18 | 281, 284, 287, 293 | 281, 287 |
|  |  |  | R-GCCTTGGAATAAACAGATTTGG |  |  |  |  |  |
| VCB-C-00694 | (gtg)5 | Y | F-GTGCCAAAGTTCAAAATTCTCC | 300 | Vitis vinifera (Grape)Whole genome shotgun sequence of line PN40024, scaffold_40.assembly12x | 2E-37 | 318, 321, 324, 328 | 328, 336, 346 |
|  |  |  | R-GATGTTGAAACAGGATTAGGGC |  |  |  |  |  |
| VCB-C-03534 | (ag)7 | N | F-AATTGCACAACCATTTCGCT | 300 | Mus musculus (Mouse)3'-5' exoribonuclease CSL4 homolog | 5E-14 | NA | NA |
|  |  |  | R-CAACCATATTTTTGCAGCAGTC |  |  |  |  |  |
| VCB-C-06669 | (tttc)3 | N | F-CATGAGTGGGGTAAGAAGAAGG | 300 | Populus trichocarpa (Western balsam poplar) (Populus balsamifera subsp. trichocarpa)Predicted protein | 6E-21 | 321, 323 | 319, 323 |
|  |  |  | R-CCCTACAGCATAAACGGGTTAG |  |  |  |  |  |
| VCB-C-07477 | (ggt)5 | N | F-GCTATGAGACAGGGAGAGAGGA | 300 |  |  | 417, 425, 428, 434 | 417, 425, 428, 434 |
|  |  |  | R-AAAGCAAGCTAGCCTTTGAATG |  |  |  |  |  |
| VCB-C-07969 | (tc)5 | Y | F-GATGCTGATTATGCTGGTGTGT | 300 | Drosophila melanogaster (Fruit fly)Copia protein | 2E-22 | 320 | 313, 320 |
|  |  |  | R-CAATATGTTTGGAGCGTGAAGA |  |  |  |  |  |
| VCB-C-09467 | (gtg)4 | Y | F-ATCAAGTACATCATCACAGCCG | 300 | Nicotiana tabacum (Common tobacco)Multi antimicrobial extrusion family protein | 6E-18 | 307, 310, 313, 316, 319, 321 | 307, 313, 321 |
|  |  |  | R-CGAACCATCCTTGTTGTACTCA |  |  |  |  |  |
| VCB-C-10738 | (ga)5 | Y | F-AGGGTTTAGGGTTTTTGTCGAT | 300 | Ricinus communis (Castor bean)Expressed protein, putative | 2E-19 | NA | NA |
|  |  |  | R-CATTTGCCGATACTGATGAAAG |  |  |  |  |  |
| VCB-C-12112 | (agg)4 | Y | F-TTCTTACCCCATAAATGCAAGG | 300 |  |  | NA | NA |
|  |  |  | R-TTACAACCGTAACGAAGTGTGG |  |  |  |  |  |
| VCB-C-12195 | (tg)6 | N | F-TCCTGGTCGGAGTAGTTTGATT | 300 | Vitis vinifera (Grape)Putative uncharacterized protein | 6E-19 | 321, 329, 332, 338, 343, 367 | 332, 338, 340, 343, 345 |
|  |  |  | R-CAGCAACAGCAGATGTATTTCC |  |  |  |  |  |
| VCB-C-13913 | (ct)5 | N | F-TTTCCATGAGGAGGAGAACATT | 300 | Ricinus communis (Castor bean)Putative uncharacterized protein | 9E-103 | 314, 321 | 319, 321 |
|  |  |  | R-GATGGGATAAAGTTGGAGCAAA |  |  |  |  |  |
| VCB-C-14219 | (ga)9 | N | F-ACTTCCATCATTTTCCACATCC | 300 | Arabidopsis thaliana (Mouse-ear cress)Glutaredoxin-C1 | 2E-37 | NA | NA |
|  |  |  | R-CAACACCTGGTGCACTTCTTT |  |  |  |  |  |
